# Research progress in isolation and identification of rumen probiotics

**DOI:** 10.3389/fcimb.2024.1411482

**Published:** 2024-05-21

**Authors:** Runmin Wu, Peng Ji, Yongli Hua, Hongya Li, Wenfei Zhang, Yanming Wei

**Affiliations:** College of Veterinary Medicine, Gansu Agricultural University, Lanzhou, China

**Keywords:** rumen probiotics, microorganisms, isolation, culture, identification

## Abstract

With the increasing research on the exploitation of rumen microbial resources, rumen probiotics have attracted much attention for their positive contributions in promoting nutrient digestion, inhibiting pathogenic bacteria, and improving production performance. In the past two decades, macrogenomics has provided a rich source of new-generation probiotic candidates, but most of these “dark substances” have not been successfully cultured due to the restrictive growth conditions. However, fueled by high-throughput culture and sorting technologies, it is expected that the potential probiotics in the rumen can be exploited on a large scale, and their potential applications in medicine and agriculture can be explored. In this paper, we review and summarize the classical techniques for isolation and identification of rumen probiotics, introduce the development of droplet-based high-throughput cell culture and single-cell sequencing for microbial culture and identification, and finally introduce promising cultureomics techniques. The aim is to provide technical references for the development of related technologies and microbiological research to promote the further development of the field of rumen microbiology research.

## 1 Introduction

The Food and Agriculture Organization of the United Nations and the World Health Organization defines probiotics as live microorganisms which provide a beneficial effect on the host when ingested in moderation (FAO/WHO, 2001). This definition is widely accepted by the International Scientific Association for Probiotics and Prebiotics (ISAPP) (Hill et al., 2014). Probiotics perform many biological functions in the ecosystem, such as aiding in digestion, inhibiting pathogenic bacteria, promoting growth and regulating immunity (Dasriya et al., 2024). The efficacy of probiotics has been demonstrated through *in vivo* experiments. Probiotics’ beneficial effects and safety are usually evaluated through *in vivo* experiments, while their beneficial potential and safety are characterized through *in vitro* studies or animal models (Reid, 2006). Therefore, the first step in evaluating probiotics for food use is *in vitro* studies, followed by double-blind, randomized, placebo-controlled human trials (Fijan, 2014; de Melo Pereira et al., 2018).General *in vitro* study properties of probiotics include resistance to gastric acid, bile acid resistance, adhesion to mucus or human epithelial cells, antimicrobial activity against potentially pathogenic bacteria or fungi, reduction of pathogen adhesion, bile salt hydrolase activity and enhancement of beneficial bacterial viability (Megur et al., 2023; Prakash et al., 2023; Sreepathi et al., 2023).

The rumen is one of the vital nutrient digestive organs in the digestive system of ruminants, and its internal microflora is very rich, including bacteria, fungi, archaea and a small number of phage viruses (Mizrahi et al., 2021). Rumen probiotics are probiotics that grow and multiply in the rumen of ruminants (Kmet et al., 1993) Rumen probiotics have specific functional roles in nutrition, digestion, immunity and health of ruminants. Numerous studies have shown that rumen probiotics can promote nitrite metabolism (Latham et al., 2018; Deng et al., 2021) promote intestinal development (Arshad et al., 2021) reduce methane production (Maake et al., 2021; Pittaluga et al., 2023), Fibre degradation (Chen et al., 2022), inhibition of pathogenic bacteria (Poothong et al., 2024), immunomodulation (Varada et al., 2022) and regulation of rumen acidosis (Lettat et al., 2012).

**Figure 1:**
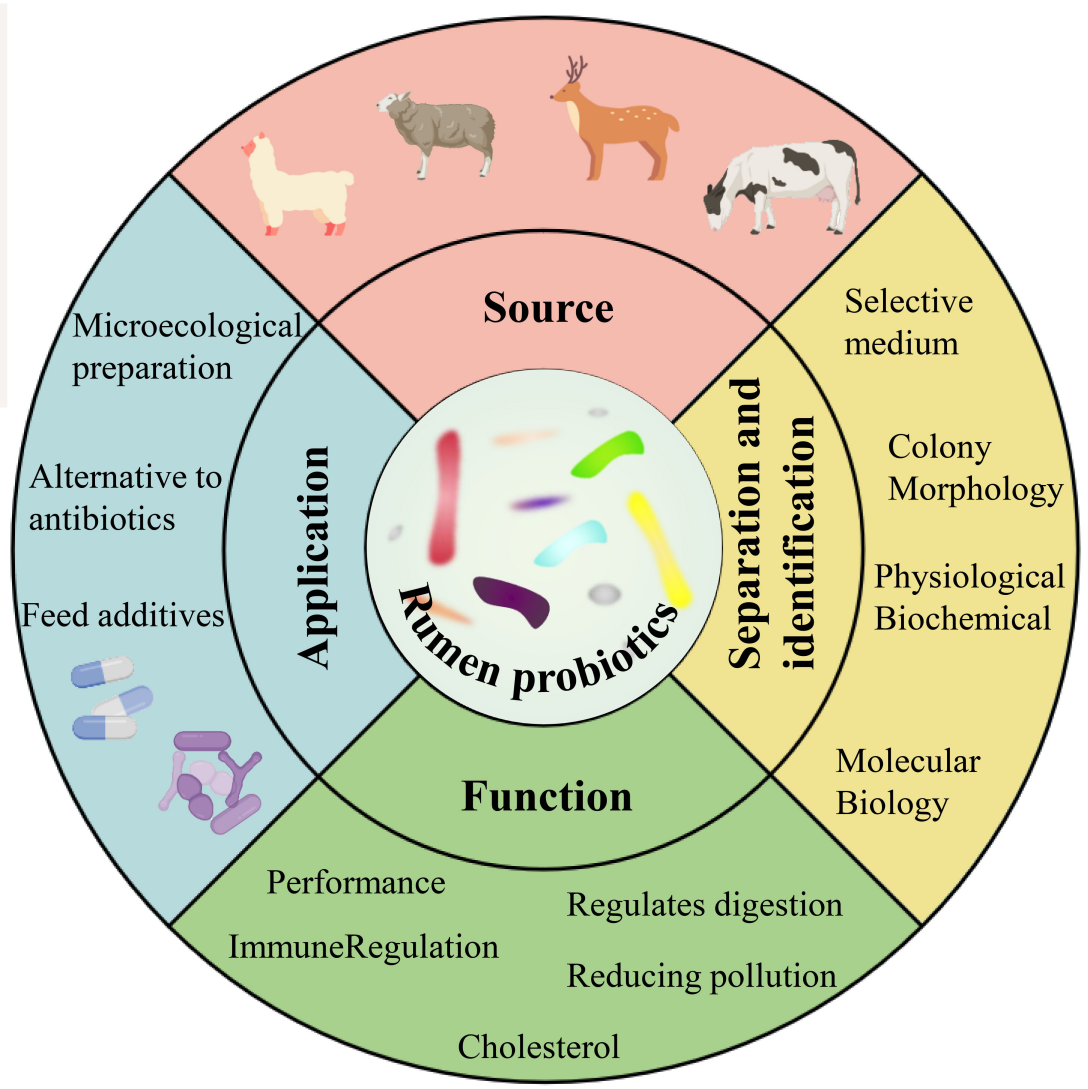
Sources, isolation and identification, functions and applications of rumen probiotics (Yuan et al., 2015; Noel et al., 2019; Xiong et al., 2020).

In recent years, high-throughput sequencing has provided a wealth of information on the composition, host specificity, and spatial and temporal dynamics of rumen-associated microbial communities (Li et al., 2023c). With the development of non-targeted and new high-throughput culture methods, culture genomics platforms using a range of media and high-throughput screening methods offer the potential to bring more “dark matter” into culture (Zhang et al., 2021). The genomics-based approach provides additional insights and suggests new hypotheses for most uncultured organisms (Gutleben et al., 2018). More importantly, with the elucidation of the beneficial mechanisms of some new strains, mining the next generation of candidate probiotics from the gut has become a new research hotspot (Wan et al., 2023). This paper provides an overview of the current applications of rumen probiotics in production, the classical techniques for culturing and identifying rumen probiotics, the culture strategies for cultivating “dark matter” from the rumen, and the technological tools for analyzing the diversity and dynamics of rumen bacteria (**Figure 1**).

## 2 Probiotic bacteria in livestock and poultry breeding applications

With the current research background of “forbidden resistance” on the feed side and “antibiotic reduction” and “substitute antibiotic” on the breeding side, probiotics are often used in the most common and widely used feed additives in the market because they can effectively improve the growth performance of livestock, enhance the immunity and regulate the gastrointestinal flora, and they are safe and environmentally friendly (Li et al., 2019; Wang et al., 2019a). Probiotic microecological preparations are prepared by isolating, identifying, and screening probiotics and their metabolites from different ecosystems and evaluating their safety *in vitro*, and through a series of technological treatments, they are prepared into biological preparations containing rich active bacteria (Xie et al., 2021). Current research has shown that enzyme preparations of cellulolytic bacteria isolated from the rumen of reindeer can increase average milk yield, enhance ciliate abundance, and improve the natural resistance of newborn calves (Litonina et al., 2021); The addition of Lactobacillus casei and Saccharomyces cerevisiae to diets increased the growth performance and apparent digestibility of nutrients, the relative abundance of short-chain fatty acid-producing microbiota, and the total short-chain fatty acid content of fattening pigs (Hassan et al., 2020). A strain of Bacillus denitrificans 79R4 (Paenibacillus 79R4) isolated from the bovine rumen enhanced rumen nitrite detoxification and reduced the risk of methemoglobinemia in cattle due to nitrate addition (Latham et al., 2019).

Other selected probiotic or potential probiotic application studies are shown in **Table 1**.

**Table 1:** Research on the application of some probiotics in livestock and poultry farming.

Probiotics	Animals	Functionality	Reference
Bacillus paralicheniformis (SN-6)	Simmental beef cattle	Increases body weight, alters metabolomic patterns in Simmental beef cattle, and increases the relative abundance of beneficial bacteria. Enriches intestinal metabolites to maintain intestinal homeostasis. Enhances amino acid metabolism and lipid metabolism pathways	Yang et al., 2022
Lactobacillus plantarum	Ovine	Increased digestibility and reduced methane emissions	Zhang et al., 2022
Lactobacillus acidophilus	Ovine	Reducing Salmonella carriage in sheep	Pepoyan et al., 2020
Bacillus subtilis	Infected computer in a botnet	Improve broiler growth performance and enhance intestinal immunity	Khalifa et al., 2023
Bacillus licheniformis	Ovine	Reducing methane emissions from sheep while increasing ration conversion rates	Deng et al., 2018
Lactobacillus rhamnosus	Piglet	Improves the physical, biological and immune barrier of the intestinal mucosa and benefits the intestinal health of pre-weaned piglets	Wang et al., 2019b
Enterococcus faecalis	Milk cow	Its secretion produces the peptide AS-48, which is used in the prevention and treatment of mastitis in cows	Davidse et al., 2004
Enterococcus faecium	Pigeon	Improve antioxidant performance and immune function of pigeon, promote growth and improve meat quality	Han et al., 2022
Ruminococcus flavefaciens	Lambs	Increases daily lamb weight gain and nutrient digestibility, reduces NH3 -N and methane production, and reduces greenhouse gas emissions	Kumar et al., 2021
Clostridium butyricum	Goats	Improving rumen fermentation and growth performance of goats under heat stress	Cai et al., 2021
Bacillus amyloliquefaciens (H57)	Ovine calf	Affects animal behaviour, promotes digestion and increases body weight	Schofield et al., 2018
Pichia kudriavzevii	Mongolian gazelle	Enhanced acetate-based fermentation for fibre and lipid digestion	Wang et al., 2022

## 3 Isolation of rumen probiotics

**Figure 2:**
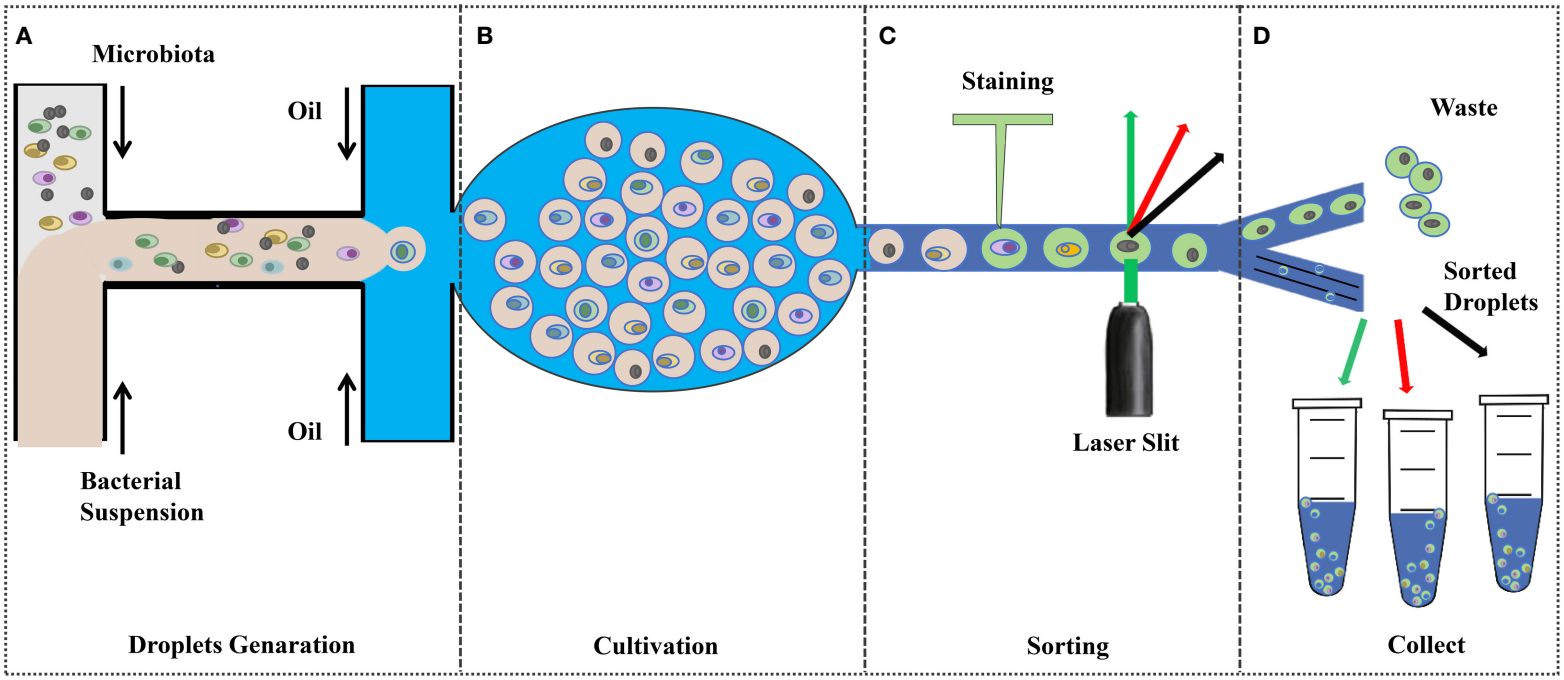
Droplet-based high-throughput culture (Tan et al., 2020; Anggraini et al., 2022; Yin et al., 2022; Zhao et al., 2016). **(A)** Pre-treated bacterial suspensions were injected into the microfluidic device to encapsulate the cells in the droplets; **(B)** Incubation under ideal conditions; **(C)**;Fluorescence Signal Dependent Droplet Sorting; and **(D)** collect.

The growth of microorganisms depends on various nutrient factors within the medium and various physicochemical factors in the culture environment, which are the primary conditions for the pure culture of microorganisms Ren et al. (2016). Therefore, understanding the nutritional requirements and metabolic properties is very important for the isolation and stable cultivation of microorganisms. The pure culture of rumen microorganisms is an important cornerstone for in-depth research on genes, proteins, and metabolic pathways, as well as a valuable resource for experimental research on microbial traits, enriching reference databases and biological classification frameworks (Stewart et al., 2019). The classical methods for isolation and culture of rumen probiotics include selective isolation and culture and the Heinz rolling tube method, which have inherent drawbacks such as low throughput, labor-intensive and high cost. The development of emerging technologies, such as microfluidic devices, offers great promise for high-throughput cultures (**Figure 2**).

### 3.1 Selective isolation culture method

Selective isolation and culture method is to prepare the corresponding selection medium to enrich the target probiotics, which is essentially to eliminate the undesired microbial flora (Bonnet et al., 2019).The media used can be undefined and optimize the media using biochemical studies or one-factor methods according to experimental needs, which can reduce the complex effects between complex components and also allow easier detection of target metabolites (Hayek et al., 2019; Lewis et al., 2021). However, the number of rumen probiotics in the traditional culture strategy is limited, and the growth and metabolic characteristics of many rumen microorganisms are still unclear, which hinders the systematic study of rumen microorganisms. Currently, selective culture media among probiotics include Bifidobacterium selective medium, Lactobacillus selective medium, low carbon-resistant selective medium, and sodium carboxymethylcellulose, etc (Kumar et al., 2021; Jaglan et al., 2019; Kuppusamy et al., 2020). The addition of antioxidants (ascorbic acid, glutathione, and uric acid in the culture medium) enhances the culture of anaerobic bacteria (Tidjani Alou et al., 2020); It can also use the rumen fluid to simulate the natural environment of certain bacteria to promote their growth (Bonnet et al., 2019). Recently, strategies combining classical microbiological techniques with macro-barcoding methods have emerged to assess the selective enrichment efficacy of media for specific rumen microorganisms (Botero Rute et al., 2023). In addition, pharmaceuticals can be added to the medium as screening indicators according to experimental needs, e.g. Tyagi et al. (2020) to obtain lactic acid bacteria with conjugated linoleic acid production potential from the rumen fluid of lactating goats, linoleic acid was added to MRS broth medium; Zhong et al. (2023) to isolate novel urea-hydrolyzing bacteria from the rumen fluid of dairy cows, urea and phenol red were used as the screening indexes.

### 3.2 Hungate’s roll-tube technique

This method was proposed by American microbiologist Hungate in 1950 and applied to the isolation and culture of anaerobic bacteria (Hungate and Macy, 1973). After years of practice and improvement has been very mature. The basic steps of this method4~5 mL of dissolved agar culture solution were dispensed into Heinz tubes and inoculated when it cooled down to 43°C, immediately, the tubes were rolled rapidly at low temperatures to solidify the liquid agar. After agar curing, microbial colonies will be produced after several days of incubation under a constant temperature incubator at 39°C. Then, under an anaerobic environment, single colonies on the wall of the rolled tubes will be picked out by inoculation ring and put into fresh medium, and purified strains will be obtained through several times of rolling tubes and isolation of single colonies and the number of viable bacteria in the bacterial liquid will be measured, which will provide a basis for quantitative determination (Cheng et al., 2006; Jing et al., 2011). The Heinz tube rolling method can meet the growth requirements of different cultures, and isolation, purification, and morphological identification can be achieved in one tube. However, different microorganisms have different needs for the optimal medium, resulting in different growth rates, which has a greater impact on quantitative analysis (Guo et al., 2009). Hu et al. (2022) used the roll-tube technique to isolate Streptococcus equinus, Enterococcus avium, and Streptococcus lutetiensis, which have inhibitory effects on Escherichia coli and Staphylococcus aureus, from the rumen juice of Holstein cows as potential probiotics or silage inoculants. Kumar et al. (2021) screened three strains of cellulose-degrading rumen-producing Ruminococcus flavefaciens from buffalo rumen fluid by dispensing carboxymethylcellulose medium in Heinz tubes, and subsequent animal experiments showed that Ruminococcus flavefaciens significantly increased the number and activity of beneficial gut microorganisms and enhanced the digestive function of milk-producing buffaloes.

### 3.3 Droplet-based single-cell cultures

For microbial culture and screening, droplet-based microfluidic methods offer the advantages of single-cell, high-throughput, high-resolution, and low-cost, making them a promising approach for isolating uncultured microorganisms (Huys and Raes, 2018). Droplet-based microfluidics can provide a separate space for cells to divide and block the influence of competitors or predators, precisely control the microenvironment of single-cell cultures, manipulate individual cells through external interventions, and detect single-cell behaviors in real time for enzyme, antibody, or rare-cell screenings, and recover cells from the microfluidic system for a variety of downstream analyses, even after cultivation (Huys and Raes, 2018; Li et al., 2023a).

Microfluidic Stripe Plate (MSP) and SlipChip are two representative microfluidic methods for static droplets (Yu et al., 2022). MSP builds on traditional streak plate technology for high-throughput single-cell analysis and culture using nanolitre droplets that can be manually or robotically streaked onto Petri dishes for single-cell culture, as well as encoding chemical gradients in the droplet array for comprehensive dose-response analysis (Jiang et al., 2016). MSP can restore microbial diversity more than traditional agar plates. Currently, there are examples of applications of the MSP method in culturing and isolating Fastidous bacteria, such as the successful isolation of fluoranthene-degrading Blastococcus from the soil, studies of termite gut microbiota, and isolation of rare deep-sea biosphere members in long cultures (Jiang et al., 2016; Xu et al., 2018a; Zhou et al., 2019a). The SlipChip consists of two glass plates with several pre-filled reagent holes, the top plate is pre-filled with samples, and the bottom plate is pre-filled with reagents. Fluorocarbon compounds act as a lubricant layer between the two plates, and when the two plates slip, the complementary pattern of holes in the plates overlap to form tens of thousands of closed chambers or channels, the top plate sample-containing wells are exposed to the bottom plate reagent wells for reaction (Du et al., 2009). The enclosed microenvironment is particularly suitable for the cultivation of anaerobic microorganisms (Yu et al., 2022). Chen et al. (2019) A SlipChip device for chemotaxis sorting and a microfluidic streak plate device for bacterial culture were newly developed as new pipelines for screening and isolating microbial species that can degrade imidazolidinone as an imidazolidinone degrader.

Recently, Watterson et al. (2020) built a platform consisting of an image processing system and a droplet microfluidic device operating in an anaerobic chamber. This platform sorts slow-growers in microorganisms in droplets by density, speeding up their growth and enriching rare taxa in fecal microbiota samples,and realize high-throughput single-cell cultivation. However, as the vast majority of microorganisms colonizing the gastrointestinal tract are almost exclusively anaerobic, integrating a single-cell isolation platform into a standard anaerobic workstation is costly. Yin et al. (2022) improved a simple droplet-based method for isolation and enrichment of functional gut bacteria by encapsulation of single-cell suspensions, which was accomplished by using diluted bacterial suspensions as the dispersed phase mineral oil as the continuous phase and then transferring to agar plates in an anaerobic chamber for incubation to form discrete single-cell colonies. This method does not require sophisticated instrumentation to sort droplets and therefore can easily be operated in a conventional anaerobic chamber to successfully isolate anaerobic Lactobacilli and Bifidobacteria.

### 3.4 Droplet-based co-culture techniques

Droplet-based co-culture systems are likewise a promising approach for the discovery of natural microbial products and the isolation of uncultured microorganisms (Jian et al., 2020; Park et al., 2011). The system promotes continuous co-culture of colonies and cells by adjusting the concentration of microbial communities, developing microdroplets with different proportions of cells, simulating microenvironments to meet microbial growth requirements, and securing information exchange between strains (Qi et al., 2023). This process is expected to solve the problem that traditional pure culture methods can interrupt the ecological interactions between microorganisms, and provide a promising way to assess complex microbial communities in more detail (Anggraini et al., 2022; Kim et al., 2023; Baichman-Kass et al., 2023). Hua et al. (2022) created a rapid screening platform for actinomycetes——the whole-cell biosensor and producer co-cultivation-based microfluidic platform for screening actinomycetes (WELCOME), By combining an MphR-based Escherichia coli whole-cell biosensor sensitive to erythromycin with Saccharopolyspora erythraea co-cultivation, they isolated six high production erythromycin-producing strains from industrial strains within a short time. Tan et al. (2020) Complex human faecal samples were dissected into sub-communities for highly parallel co-culture using a droplet microfluidic device. Twenty-two individual droplets with strong bacterial symbiosis were then selected by microfluidics, in which a partial genome of a representative of a new genus of Neisseriaceae was found, highlighting the ability of microfluidic co-cultures to access and study uncharacterized microbial diversity.

## 4 Identification of rumen probiotics

In the early days, microbiological studies based on morphological features and physiological and biochemical traits provided insights into the microbial world, but today, they can only provide limited resolution (Escobar-Zepeda et al., 2015). Advances in molecular techniques have provided access to the “new uncultured world” of microbial communities. Among these techniques, polymerase chain reaction (PCR), denaturing gradient gel electrophoresis (DGGE), terminal restriction fragment length polymorphism (T-RFLP), fluorescence *in-situ* hybridization (FISH) and rRNA gene cloning and sequencing have had a significant impact (Escobar-Zepeda et al., 2015). However, while they describe the diversity of microorganisms, amplicon sequencing, macrogenomics, and single-cell genomics are the most widely used techniques for solving environmental microbiological problems (Xu and Zhao, 2018b).

### 4.1 Morphological identification

Morphological features are the important references for the classification and identification of microorganisms. Identification is carried out by observing both colony morphology and microscopic morphology. Colony morphology includes colony size, shape, surface, texture, transparency, degree of elevation, and medium color (Meruvu and Harsa, 2023). The bacterial morphology reflects the survival value of bacteria for acquiring nutrients, moving, and avoiding predators (Young, 2007). In microscopic morphology, commonly used types of microscopes are ordinary optical microscopes and phase contrast microscopes, for ultra-fine structures and complex structures can use scanning electron microscope and transmission electron microscope observation (Sun and Lin, 2009). With the rapid development of microimaging technologies and microfluidic chips, it becomes feasible to observe microbial germination and growth in real time (Zhou et al., 2019b). Zhang et al. (2019) used a gradient microfluidic chip to observe the morphological changes of various bacteria under the action of antibiotics. The rumen probiotics can also be classified with the help of Gram staining, which provides a reference basis for isolating rumen probiotics (Wang et al., 2021). Han et al. (2018) and Li et al. (2017) used colony morphology and Gram staining techniques for preliminary isolation and identification of probiotic bacteria in the samples, followed by 16SrRNA gene homology analysis to obtain probiotic strains of bovine origin.

### 4.2 Physiological and biochemical tests

The production and metabolic activities of microorganisms depend on extracellular enzymes to degrade macromolecular substances, the enzyme systems of different microorganisms will show significant differences in metabolic activities, which are confirmed by the changes in the substances in the vicinity of the microbial colonies, providing a basis for the identification and classification of microorganisms (Wu et al., 2018). At present, the physiological and biochemical tests that are more frequently used in identifying probiotics include the gelatin liquefaction test, methyl red test, glycolysis test, indole test, and starch hydrolysis test for the preliminary assessment of probiotics (Lin, 1999). The test is used for the preliminary evaluation of probiotics. However, due to the slow growth of some bacteria and the difficulty of cultivation, they cannot meet the requirements of biochemical reactions for microbial concentration and colony freshness, and the accuracy of biochemical tests for identifying anaerobic microorganisms in the rumen needs to be improved. Abid et al. (2022) in order to screen probiotic strains favorable for milk fermentation, four strains of Lactobacillus spp. were screened from 15 strains showing gas production in Durham tubes. These four probiotic strains were identified morphologically, identified by biochemical tests, and evaluated for their probiotic potential. It was found that Lactobacillus fermentum strains showed significant viability in the presence of pepsin, trypsin, and lysozyme.

### 4.3 16SrRNA-based amplicon sequencing

The advantage of amplicon sequencing lies in the contrasting biases generated by using only one phylogenetic marker (Escobar-Zepeda et al., 2015). Because 16SrRNAs are ubiquitous in all species and are functionally integral to the core genome, the composition and relative abundance of microbial communities in environmental samples are often investigated by amplifying and sequencing specific regions of the 16SrRNA gene (Daubin et al., 2003; Větrovský and Baldrian, 2013). The 16SrDNA gene sequence is divided into constant and variable regions. The constant region reflects the kinship between species (Head et al., 1998); The variable region reflects the specificity of the species and is used to classify them biologically (Chaudhary et al., 2015). Second-generation sequencing platforms, such as Illumina, can sequence amplicons of up to 600 bp with high precision (Bharti and Grimm, 2021). Third-generation amplicon sequencing platforms such as PacBio and Oxford Nanopore can sequence full-length 16S rRNA genes in a short period of time at the single-molecule level, which reduces the problems of amplification bias and short read lengths and makes it possible to annotate the microbiome at the species and strain level (Abellan-Schneyder et al., 2021). The 16SrRNA homology analysis has been successfully used to construct a gene library of Holstein cow rumen bacteria, which facilitates microbial species analysis (Yang et al., 2010; King et al., 2011). Although the 16SrRNA identification technique reflects the diversity of rumen microbes, it does not have a sufficient resolution at the species level, resulting in a loss of information on low-abundance members of the microbiota and an inability to understand the function of the community (Bowers et al., 2022). Abedini et al. (2023) reported for the first time the isolation of probiotics from camel rumen, preliminary screening of Gram-positive, catalase-negative colonies with white-colored colonies from the contents of camel rumen, identified as Enterococcus faecium96B4 by 16SrRNA, and subsequent evaluation of the probiotic activity and safety evaluation revealed good probiotic potential, reflecting the potential research value of camel rumen as a pristine environment.

### 4.4 Denaturing gradient gel electrophoresis

DGGE is an electrophoretic technique that separates DNA fragments based on differences in the order of DNA bases and is used to detect nucleic acid mutations and point mutations. Its basic principle is that in DNA molecules under the influence of specific temperature conditions and chemical denaturants, a region of double-stranded DNA starts to unlink, the unlinking region is related to the order of the base arrangement, and the unlinking can occur with the difference of only one base pair, and the difference of the base sequence of the DNA fragments will be denatured under the corresponding denaturing conditions in the process of the denaturing gradient gel swimming. When the ends of double-stranded DNA molecules are unstranded, their electrophoretic resistance increases greatly and their speed decreases significantly, which leads to the DNA fragments staying in different parts of the gel to achieve separation (Li et al., 2008; Ercolini, 2004). DGGE has the characteristics of good reproducibility, high detection rate, convenience and quickness, etc. It can be applied to the analysis of uncultured microorganisms (McGenity et al., 2010). DGGE was first applied to the structural analysis of rumen microorganisms by Kocherginskaya et al. (2001) analyzed the effect of two diets, hay, and maize, on the structure of the rumen bacterial community of castrated cows and showed that the bacterial populations in the rumen were relatively more diverse and numerous after feeding the maize diet. Through DGGE technology, Yu et al. (2020) found that supplementing Dihydropyridine (DHP) in the diet can promote the growth of Xanthomonadaceae and Xanthomonas and enhance the diversity of ruminal bacteria. Min et al. (2021) explored the effect of supplementing Condensed Tannins (CT) on calf rumen bacterial diversity and methane emissions. DGGE results showed that Firmicutes and Bacteroidetes populations seemed to increase as CT content increased., CT and can exert anti-methanogenic activity by directly inhibiting methanogens or indirectly through rumen fermentation, thereby reducing methane emissions.

### 4.5 Terminal restriction fragment length polymorphism

T-RFLP is an extension of RFLP. T-RFLP allows culture-independent assessment of subtle genetic differences between strains and provides a molecular approach to the evaluation of microbial community structure and function (Marsh, 1999). The technique uses PCR to amplify small subunit rRNA genes from total community DNA, where one or two primers are labeled with a fluorescent dye, followed by digestion of the PCR product with a restriction endonuclease with a four-base-pair recognition site and determination of the size and relative abundance of fluorescently-labeled T-RFs using a DNA sequencer. Because differences in T-RF size reflect sequence polymorphism, phylogenetically distinct populations of organisms can be resolved (Schütte et al., 2008). Although the use of T-RFLP is declining, it is still the method of choice for community dynamics studies (Prakash et al., 2014). Zhu et al. (2018) combined T-RFLP and clone library analysis to compare changes in rumen bacterial and archaeal communities in response to dietary disturbances before and after low-grain and high-grain production and found significant changes in the relative abundance of methane-producing communities in cows during the transition period, as well as a clear shift in rumen fermentation patterns.

### 4.6 Fluorescence *in situ* hybridization

FISH was introduced in 1980 (Bauman et al., 1980). FISH uses fluorescein oligonucleotide probes to bind complementarily to specific target nucleic acid sequences and detects the corresponding fluorescent signals for single-cell identification and quantification by fluorescence microscopy, whole-slide images, or flow cytometry (Amann and Fuchs, 2008). FISH has been widely used for the diagnosis of chromosomal aberrations in medicine, the identification of microorganisms in complex samples, and the identification of microorganisms (Ratan et al., 2017)., also provides a basis for *in situ* image-based spatial transcriptomics (Wen et al., 2022; Zhou et al., 2023). With improvements in fluorescence microscopy and fluorescent labeling of various nucleic acid probes, FISH has evolved to be used with other biotechnologies as a rapid and accurate biosensor system (Kuo et al., 2020). FISH combined with Raman spectroscopy can rapidly identify target microorganisms in complex samples by labeling the DNA of specific species (Cui et al., 2022). FISH can also be integrated into microfluidic microarray platforms to speed up the process of colony identification, reduce reagent consumption, and have the potential for automation (Rodrigues et al., 2021). Liu et al. (2009) developed a microfluidic device that integrates FISH identification and droplet-splitting modules for parallel high-throughput single-cell culture and identification. The single-cell droplet was split into two sub-droplets with the aid of a droplet-splitting chip, one of which was added to an agar plate, and the other was subjected to FISH identification, and the droplet that encapsulated the target species was finally selected from the agar plate based on the identification results. Liu et al. (2011) Seamlessly integrated two components, FISH and fluorescence-activated cell sorting (FACS), into a microfluidic device, which forms a hybridization chamber between two photopolymeric membranes in which cells and probes are electrophoretically loaded, incubated, and washed; a downstream cross structure is used to electrically focus the cells into a single flow for FACS analysis, providing a quantitative detection of microbial cells in complex samples automated platform. Batani et al. (2019) developed a new method to isolate live bacteria based only on their 16S rRNA gene sequences (Live-FISH), which combines FISH with FACS to enable the sorting and culturing of live bacteria. With the development of highly specific probes, Live-FISH has greater potential for targeted sorting of target microorganisms. However, FISH requires the rRNA content or the number of microorganisms in the probe target organisms, otherwise the fluorescence signal cannot be detected under the microscope (Hoshino et al., 2008). In addition, the FISH technique suffers from the problem of crosstalk of organic fluorescein excitation light (Waters, 2009). Meanwhile, FISH can only identify a small number of microorganisms with a high degree of certainty in a single experiment, and cannot elucidate the distribution of microorganisms in the overall microecosystem. The development of combinatorial labeling and spectroscopic imaging can increase the microspatial relationships of different species of microorganisms in a single field of view, but it is too costly (Valm et al., 2011).

### 4.7 Macrogenomics

Automated sequencing of DNA by Sanger sequencing in the late 1970s ushered in the era of genomics Sanger sequencing retrieves up to 96 sequences per run at an average length of 650 bp, which may be sufficient for phylogenetic marker analyses, but the emergence of what is known as next-generation sequencing (NGS) technology has enabled researchers to bypass the cultivation of parallel sequencing of millions of DNA molecules with varying yields and sequence lengths directly after extraction from highly diverse populations, i.e., macrogenome sequencing (Escobar-Zepeda et al., 2015; Hu et al., 2021). It is usually performed using birdshot sequencing methods that are non-discriminatory, allowing for the quantitative assignment of taxonomy and organisms to the species level, and with the help of databases of identified functional genes, such as KEGG, GO, COG, and eggNOG, the understanding of microbial communities in terms of their composition, function, evolution, and interactions in their natural environments, which directly contributes to the flourishing of microbial ecology (Tatusov et al., 1997; Kanehisa et al., 2008; Huerta-Cepas et al., 2016). However, macrogenome sequencing uses environmental DNA samples, which cannot link each functional gene to a specific microbial individual, and for high-diversity samples or low-abundance organisms, it is very difficult to assemble a single discrete genome to capture strain-level variation (Kaster and Sobol, 2020; Daliri et al., 2021). Li et al. (2023c) studied changes in bovine rumen microbes from pre-transport to 1-month post-transport by macrogenomics and found that the abundance of rumen bacteria and archaea was higher on day 16 post-transport than pre-transport, but eukaryotic abundance was highest on day 30 post-transport. Before transport, most bacteria were mainly involved in polysaccharide digestion. On day 4 post-transport, KEGG pathway enrichment was most notable for nucleotide metabolism. On day 16 post-transhipment, energy metabolism and rumen content of MCPs and VFAs increased significantly, but at the same time, energy loss due to methane production (Methanobrevibacter) and pathogenic bacteria (Saccharopolyspora rectivirgula) together induced inflammation and oxidative stress in cattle, which is important for the establishment of new management and nutritional specification strategies.

### 4.8 Single-cell genomics

Genomics for microorganisms in the environment, macro-genome sequencing describes the full range of genetic information, and single-cell genomics reveals individual genomes, and combining the two can compensate for their respective shortcomings (Nobu et al., 2015; Mende et al., 2016). Sequencing the microbiome at the resolution of individual microorganisms effectively improves the efficiency and accuracy of obtaining genome-wide information from complex microbial communities, and also allows for the study of individual cellular behaviors underlying the complexity of microbial ecosystems (Lloréns-Rico et al., 2022; Madhu et al., 2023). Single-cell isolation is essential to performing high-throughput single-cell genomics workflows (Xu et al., 2018a). Microfluidic devices offer advantages in terms of throughput, affordability, and automation for single-cell capture and retrieval applications (Han et al., 2023). Its subsequent steps include DNA extraction, 16S rRNA gene PCR phylogenetic identification, multiple displacement amplification (MDA), library construction, sequencing, and data analysis (Xu and Zhao, 2018b; Arikawa et al. (2021) developed a framework for integrating single-cell genomics and macrogenomics, integrating single-cell amplified genomes (SAG) and macrogenome assembled genomes (MAG) to reconstruct competent microbial genomes, and achieving high-quality recovery of strain-resolved genomes. Zheng et al. (2022) developed and validated a strain-resolved, high-throughput single-cell sequencing method (Microbe-seq) that uses a microfluidic platform to individually encapsulate microorganisms into droplets where whole-genome amplification and specific barcode coding are performed, followed by sequencing of merged labeled DNA to generate a single amplified genome (SAG), and then finally co-assembling the SAGs of the same bacterial species. SAGs from the same bacterial species are finally co-assembled to achieve single-strain level resolution. However, SAGs can introduce chimeric and biased sequences during genome amplification, leading to sequence incompleteness. To address this problem, Kogawa et al. (2023) developed a single-cell amplified genome long read-length assembly workflow to construct complete circular SAGs (cSAGs).

## 5 Culturography

**Figure 3:**
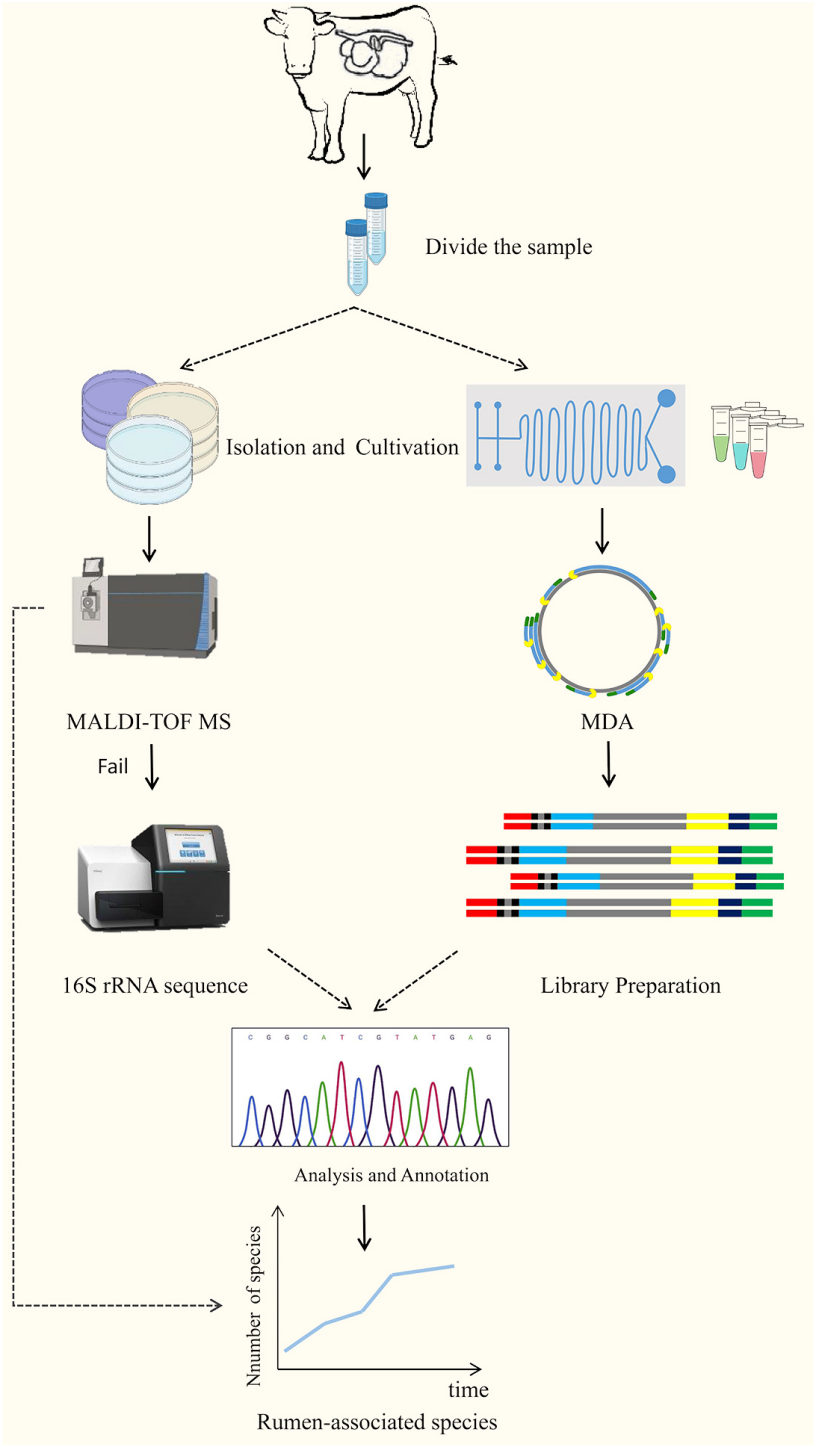
The technical route of culturomics (Wan et al., 2023).

Culturomics is a high-throughput method of isolating and culturing bacteria under as many combinations of culture conditions as possible, using a combination of matrix-assisted laser-resolved ionization time-of-flight mass spectrometry (MALDI-TOF MS) or other sequencing technologies for microbial identification (Diakite et al., 2021; Wan et al., 2023; Yu et al., 2023). It is a high-throughput method for bacterial isolation and culture. The technique was first used for the analysis of uncultured microorganisms identified in the human gut, and with the significant expansion of strain databases, many microorganisms previously overlooked or considered unculturable have been brought into the culture (Diakite et al., 2021). Classical cultureomics is an untargeted strategy that includes steps such as sample collection, sample processing, microbial isolation, culture, identification, and preservation (Wan et al., 2023). That is, the treated samples are dispersed and cultured into different media, and then the characteristic colonies are selected for identification using MALDI-TOF MS analysis if the reference profiles are lacking, 16SrRNA sequencing is required for further identification, and if the similarity is < 98.65%, it is considered to be a new species, and then describing new species using taxonomic genomics (Lagier et al., 2018; Peng et al., 2020). In addition, with the use of membrane diffusion culture (Nichols et al., 2010; Chaudhary et al., 2019) microfluidic devices (Anggraini et al., 2022; Luo et al., 2022) FACS, FISH (Waters, 2009) and reverse genomics (Cross et al., 2019), and other high-throughput culture technologies and targeted sorting techniques, the culture results of cultureomics will be greatly enriched (**Figure 3**). Combined with full-length 16S rRNA gene amplicons and birdshot macro-genome sequencing, culture-based metabonomics (CBM) will deeply explore untapped novel bacterial resources at high resolution (Li et al., 2023b). In addition, information specific to the target microorganisms obtained in advance from literature studies or macrogenomics data can also be used to reverse-guide the isolation and culture of the target microorganisms, providing additional opportunities to obtain pure cultures (Bellais et al., 2022; Liu et al., 2022). Of course, there are still some constraints in cultureomics: it is still at the beginning stage in the cultivation of rumen microorganisms, which is labor-intensive and will result in a waste of manpower and resources (Mordant and Kleiner, 2021); gut-microbiota interactions and symbiotic relationships between microorganisms are still unclear (Yadav et al., 2022).

## 6 Perspectives and conclusions

The rumen microecological environment is extremely complex, and it is difficult to simulate its physicochemical parameters *in vitro*. Traditional *in vitro* techniques for isolation and culture of rumen probiotics have significant limitations and it is difficult to identify rare microorganisms by general identification techniques. Therefore, it is necessary to introduce new techniques for large-scale mining of probiotics in the rumen. This will help rationally define the beneficial flora, pinpoint microbial-derived metabolites with beneficial effects, and provide technical support for developing novel probiotic formulations. This is of great significance for animal nutrition and health, food safety, and ecological protection.

## 7 Future Direction

The first key feature of the state-of-the-art technologies presented in this review is high-throughput culture. Traditional laboratory microbiological cultures are usually inefficient and time-consuming, but the development of microfluidic devices has created conditions for high-throughput cultures. Therefore, instrument design, microfluidic chip design, and fabrication have become crucial. The integration of experimental functions such as dilution, separation, single-cell encapsulation, anaerobic incubation, and targeted sorting onto a small chip requires interdisciplinary collaboration, while reagent development, construction of equipment such as anaerobic devices, and sorting and collection devices requires an experienced R&D team. Second, genome sequencing at single-cell resolution is another key feature. Single-cell sequencing can reveal functional information about rare species, help understand inter-microbial or microbe-host interactions, and explore potential metabolic pathways. The development of various histological approaches will also provide information on the rumen microbiome and its metabolites, which will help guide the isolation and culture of target microorganisms. While cultureomics, with its attributes of high-throughput culture and single-cell resolution, will significantly increase the potential for isolating and culturing “dark matter” from the rumen, this endeavor has significant challenges. The adaptation of optimal media for microorganisms is time- and labor-intensive, and the design of co-culture or mono-culture systems may not be able to meet the growth needs of all microorganisms. In addition, targeted sorting devices such as FACS and FISH still suffer from problems such as crosstalk and insufficient number of stains. Overcoming these technological bottlenecks requires the unremitting efforts of the R&D team.

Rumen probiotics from cattle and sheep are relatively well documented, but there are fewer reports on probiotics from antelope, deer, and musk family sources. The rumen microbial composition is very rich and susceptible to factors such as feeding management, geographic location, and significant differences in microbial composition between individuals and species, so gastrointestinal microbiological studies in rare ruminants may yield unexpected findings. In addition, the research and application of rumen probiotics require the establishment of a standard system for probiotic isolation and culture, identification, evaluation of probiotic properties, safety evaluation, and application. Only by ensuring the maximum use of strain resources can the potential of rumen probiotics be better explored, their application in agriculture and industry be improved, and a greater contribution be made to the sustainable development of animal husbandry.

## Author contributions

RW: Writing – original draft, Writing – review & editing. PJ: Conceptualization, Investigation, Project administration, Supervision, Writing – review & editing. YH: Data curation, Funding acquisition, Resources, Writing – review & editing. HL: Conceptualization, Investigation, Writing – review & editing. WZ: Data curation, Writing – review & editing. YW: Conceptualization, Funding acquisition, Writing – review & editing.
